# Knockdown resistance (*kdr*) of the voltage-gated sodium channel gene of *Aedes aegypti* population in Denpasar, Bali, Indonesia

**DOI:** 10.1186/s13071-017-2215-4

**Published:** 2017-06-05

**Authors:** Penny Humaidah Hamid, Joko Prastowo, Anis Widyasari, Anja Taubert, Carlos Hermosilla

**Affiliations:** 1 Department of Parasitology, Gadjah Mada University, Jl. Fauna No. 2, Karangmalang Yogyakarta, Indonesia; 2 RSUP Dr Sardjito, Jl. Kesehatan No. 1, Sekip Yogyakarta, Indonesia; 3 Institute of Parasitology, Biomedical Research Centre Seltersberg, Justus Liebig University Giessen, Schubertstr. 81, Giessen, Germany

**Keywords:** *Aedes aegypti*, *Kdr*, Resistance, Denpasar, Indonesia

## Abstract

**Background:**

*Aedes aegypti* is the main vector of several arthropod-borne viral infections in the tropics profoundly affecting humans, such as dengue fever (DF), West Nile (WN), chikungunya and more recently Zika. Eradication of *Aedes* still largely depends on insecticides, which is the most cost-effective strategy, and often inefficient due to resistance development in exposed *Aedes* populations. We here conducted a study of *Ae. aegypti* resistance towards several insecticides regularly used in the city of Denpasar, Bali, Indonesia.

**Methods:**

*Aedes aegypti* egg samples were collected with ovitraps and thereafter hatched in the insectary of the Gadjah Mada University. The F0 generation was used for all bioassay-related experiments and knockdown resistance (*kdr*) assays.

**Results:**

Results clearly showed resistance development of *Ae. aegypti* against tested insecticides. Mortalities of *Ae. aegypti* were less than 90% with highest resistance observed against 0.75% permethrin. Mosquitoes from the southern parts of Denpasar presented high level of resistance pattern in comparison to those from the western and northern parts of Denpasar. *Kdr* analysis of voltage-gated sodium channel (*Vgsc*) gene showed significant association to S989P and V1016G mutations linked to resistance phenotypes against 0.75% permethrin. Conversely, *Ae. aegypti* F1534C gene mutation did not result in any significant correlation to resistance development.

**Conclusions:**

Periodically surveillance of insecticide resistances in *Ae. aegypti* mosquitoes will help local public health authorities to set better goals and allow proper evaluation of on-going mosquito control strategies. Initial detection of insecticide resistance will contribute to conduct proper actions in delaying mosquito resistance development such as insecticide rotation or combination of compounds in order to prolong chemical efficacy in combating *Ae. aegypti* vectors in Indonesia.

## Background

Dengue fever (DF) is a serious arthropod-borne arboviral infectious disease, mainly distributed in tropical regions. As reviewed elsewhere [1] endemic dengue virus transmission is reported in the Eastern Mediterranean, American, South-East Asian, Western Pacific and African regions, whereas sporadic local transmission has been reported to occur in Europe and the USA. DF is a major public health concern of Indonesia as one of the principle endemic countries within Southeast Asia. Warm tropical climate, which clearly supports *Aedes* vector life, is considered one of the main risk factors [2] and influencing further the disease burden and its continuous persistence mainly in equatorial countries. In addition, the mosquito vector, *Aedes aegypti*, efficiently transmits DF due to its anthropophilic feeding and breeding characteristics [3]. It has been reported that 71,668 DF human cases have so far occurred in Indonesia in 2015 covering 34 provinces, which means that DF has spread now into all national territories [4]. During January-February of 2016, there have been 8,487 cases with 108 mortalities and 11 provinces are categorized of being at a remarkable risk. There is still little data on the origin of *Ae. aegypti*, which contributes to the DF, spread in Indonesia [5]. Active development of national programs to promote DF elimination has been initiated since many years through community based activities such as vector control strategies and trials on virus vaccination programs. Nonetheless society activities, such as the elimination of *Ae. aegypti* larvae breeding sites become difficult especially during heavy rainy seasons in Indonesia. To date, vector control is the main tool to reduce viral transmission since antiviral drugs and vaccinations are still in progress despite of intensive research and vaccine development efforts worldwide.

Thus, *Ae. aegypti* control is still based on the use of large-scale fogging and space-spraying of insecticides, although also [6] *Wollbachia*-engineered mosquitoes have also been previously used. Alongside, also the use of larvacides containing granule-temephos (ABATE®) in combination with manual-larval inspection by governmental teams are still applied in several tropical geographical regions. Integrated social activities shall be supported by perpetual motivation to close both artificial and natural water containers to prevent mosquito breeding. Nonetheless, the difficulties arise due to opened water media such as flower vases, coconut shells, trash cans cut-wooden basins, which are frequently uncontrolled. As the majority of people are using water reservoirs to bathe, this approach has to be concomittant with changes of social behavior in replacing water reservoirs periodically or by using continuously flowing water systems.

Consequently, the high usage of insecticides has generated dependency of these compounds for regular vector control activities mainly during wet seasons. More importantly, *Ae. aegypti* resistance to common used insecticides has previously been reported from different countries worldwide such as Colombia [7], Brazil [8, 9], Grand Cayman [10], Thailand [11], India [12], Malaysia [13], Mexico [14, 15] and China [16]. Moreover, mosquito resistance related studies in central regions of Java [17] and Yogyakarta [18], Indonesia, have shown the resistance development to pyrethroids. These studies reported on the association of insecticide resistance with knockdown resistance (*kdr*) genes on voltage-gated sodium channel (*Vgsc*) of *Aedes* mosquitoes. So far, the *kdr* profiling of *Aedes* have found at least seven point mutations, which can clearly lead to reduced sensitivity of sodium voltage channels to insecticides [19]. Identification of *Ae. aegypti* showed that S989P, I1011M/V, V1016G/I, F1269C, F1534C corresponded well to this resistance development [10, 20–23]. At least two of these mutations, i.e. V1016G /F1534C, and I were responsible for pyrethroid resistance [20, 21]. However, synergistic resistance may also occur by additional point mutations in different locations of *Vgsc* genes as postulated elsewhere [24].

The most populated islands in Indonesia are Java and Bali with around 60 million of citizens and this although these regions represent less than 7% of the national territory [25]. Population density is influenced by factors such as job opportunities, infrastructures, education facilities, administrative centres and touristic attractions. Disease outbreaks and vector conditions in these areas contribute significantly to the numbers to human DF cases surveillance and national control strategies [5]. Thus, the usage of skin repellents and space-spraying insecticides is much higher in these mostly residential areas. Therefore, the distribution of allele polymorphism in *Ae. aegypti* in these urban/suburban areas is important to be investigated periodically in order to guarantee proper control programs. Additionally, vast areas of the Indonesian archipelago lead to possible variations throughout tropical areas investigated. We here present genotyping, distributions and variations of *Ae. aegypti Vgsc* genes in different areas of the city of Denpasar, Bali, Indonesia.

## Methods

### Mosquito samples

**Fig. 1:**
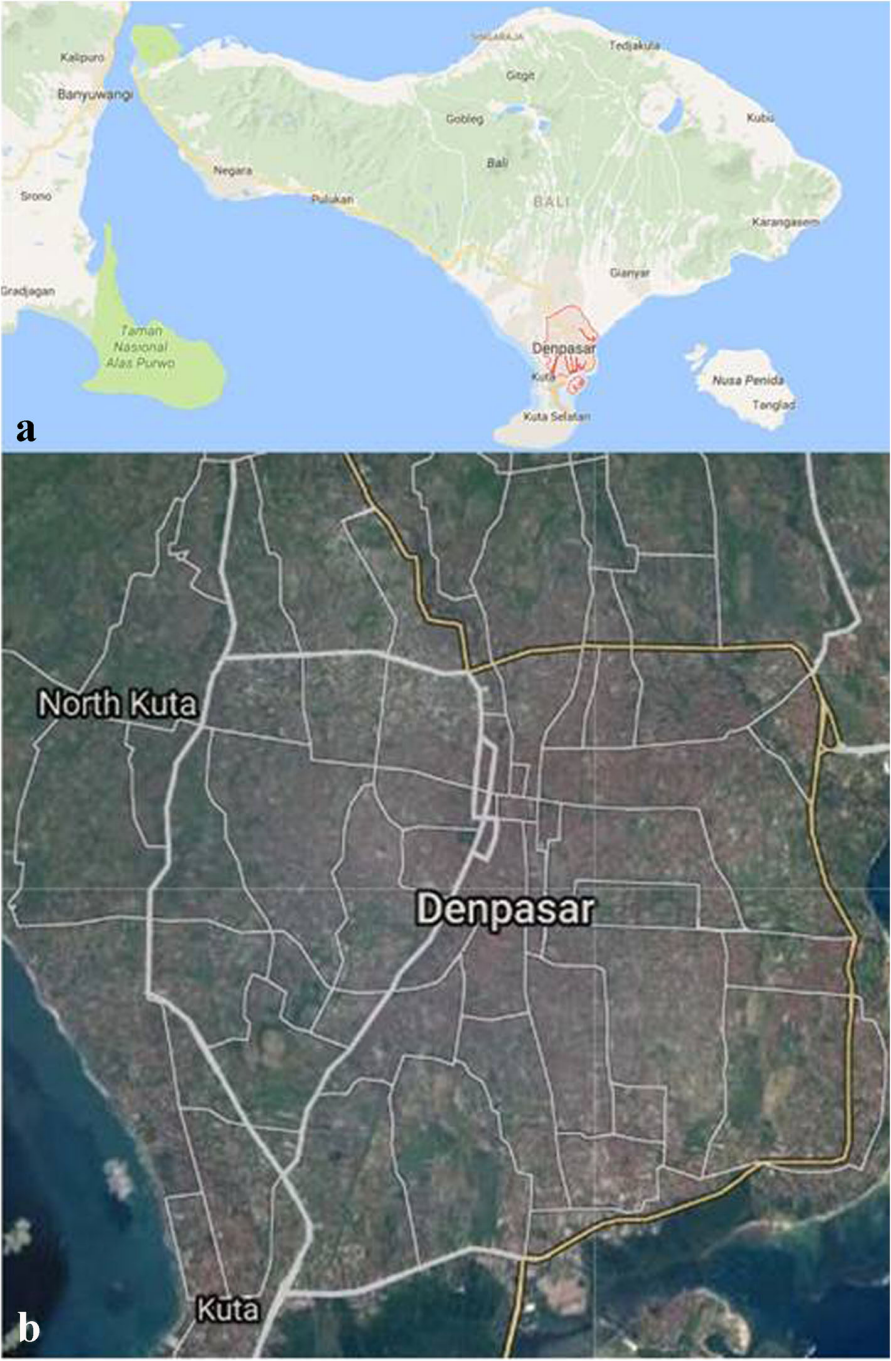
**a** Map of the Island of Bali, Indonesia. **b** Sample sites for *Ae. aegypti* egg collections within the city of Denpasar

This study was performed in Denpasar, Bali, Indonesia (Fig. 1). Southern Bali area is the most developed part of the island. *Ae. aegypti* were collected by using 300 artificial ovitraps placed in residential areas of investigated districts. Ovitraps were made of glass with black stain outside. A filter paper was placed in the mouth of the glass. Filled water and filter papers were replaced every week on collection dates. Samples were collected from April to September 2016. The collection sites were randomly selected with particular emphasis on areas with previous reports of human DF cases and in which regular fogging activities occurred. Egg-containing papers were dried at room temperature (RT) and stored in plastic containers thereafter. Eggs were hatched in the insectary of the Department of Parasitology, Faculty of Veterinary Medicine, Gadjah Mada University, Yogyakarta, Indonesia. Larvae were fed with chicken liver (wet and dried) during their development until the pupa stage. Adult *Ae. aegypti* mosquitoes reared from these collected eggs were then fed with a 10% sugar solution absorbed into cotton balls. Emerged mosquitoes (F0) up to three day-old were used for all insecticide-related experiments.

### Insecticide susceptibility tests

Insecticide succeptibility tests (IST) were performed according to the World Health Organization (WHO) protocols for anopheline mosquito’s diagnostic doses [26]. The kits and insecticide impregnated papers were supplied by the University Saints Malaysia (Penang, Malaysia). The impregnated papers consisted of 5% malathion, 0.05% deltamethrin, 0.75% permethrin, 0.05% λ-cyhalothrin, 0.1% bendiocarb and 0.15% cyflothrin, respectively. In each assay 150 adult *Ae. aegypti* mosquitoes from each areas were divided into 6 tubes containing 25 mosquitoes each. Four tubes (4 replicates) served as replicates for 1 insecticide exposure and two tubes were used as controls. The mortality was calculated as a percentage using the formula: total number of dead mosquito/total sample size × 100, as reported elsewhere [26]. Abbott’s formula was not used in this study since control mortalities were always less than 5%. Some of survivors and dead mosquitoes from the bioassays were kept at -20 °C for further molecular resistance analysis. Resistance status of mosquitoes populations were defined according to WHO as < 90% mortality as previously reported [26].

### Profiling of domains II and III of the voltage-gated sodium channel gene (*Vgs*c)

#### DNA isolation

DNA isolation was performed using PureLink® Genomic DNA Isolation Kit (Invitrogen, Carlsbad, USA) in combination with occasional vortexing using glass beads to ease mosquitoes lysis. Dead and surviving mosquitoes from different collection sites from bioassays were extracted individually.

#### Partial domain II of sodium voltage-gated channel (Vgsc) amplification

Detection of V1016G/I and S989P mutations were performed by direct sequencing utilizing primers IIP_F and IIS6_R which encompassed domain II segment 6 of *Ae. aegypti Vgsc* gene as described previously [11]. Domain II amplification was performed by using primer IIP_F dan IIS6_R (Table 1). Domain II PCR consisted of 1 μl of genomic DNA, 1 μl of 10 pmol forward primer (Macrogen, Seoul, Korea), 1 μl of 10 pmol reverse primer, 12.5 μl of DreamTaq Green PCR Master Mix (Thermo Fisher Scientific, Waltham, USA) in 25 μl total reaction volume. Both reactions were performed as follows: 95 °C for 5 min, 35 cycles of 30 s at 95 °C, 45 s at 63 °C, 45 s at 72 °C, and a final elongation step for 5 min at 72 °C. Amplified PCR product was then purified and directly sequenced (Integrated DNA Technologies/1st BASE, Singapore).

**Table 1:** Primers used in the experiments

Name	Sequence (5'–3')
IIP_F	GGTGGAACTTCACCGACTTC
IIS6_R	GGACGCAATCTGGCTTGTTA
F1534-f	GCGGGCTCTACTTTGTGTTCTTCATCATATT
C1534-f	GCGGGCAGGGCGGCGGGGGCGGGGCCTCTACTTTGTGTTCTTCATCATGTG
CP-r	TCTGCTCGTTGAAGTTGTCGAT
Ge-IIIS6_F	GCTGTCGCACGAGATCATT
IIIS6_R	GTTGAACCCGATGAACAACA

#### F1534C allele-specific PCR

Genotyping of mutant F1534C was performed according to previous publication [11] for allele - specific PCR assay. Primers used for the assay are listed in Table 1. F1534C PCR consisted of 1 μl of 10 pmol primer F1534f, 1 μl of 10 pmol primer C1534r, 0.5 μl of 10 pmol primer CPr, 12.5 μl of DreamTaq Green PCR Master Mix (Thermo Fisher Scientific) in 25 μl total reaction volume. Reactions were performed as follows: 94 °C for 2 min, 35 cycles of 30 s at 94 °C, 30 s at 60 °C, 30 s at 72 °C, and a final elongation step for 2 min at 72 °C. PCR amplification products were loaded onto a 3% agarose gel.

To confirm F1534C genotyping, several *Ae. aegypti* showing resistance phenotypes were also sequenced (*n* = 30). Amplification was performed by utilizing primers Ge-IIIS6_F and IIIS6_R [11]. Domain II PCR consisted of 1 μl of genomic DNA, 1 μl of 10 pmol forward primer (Macrogen), 1 μl of 10 pmol primer reverse primer, 12.5 μl of DreamTaq Green PCR Master Mix (Thermo Fisher Scientific) in 25 μl total reaction volume. Reactions were performed as follows: 95 °C for 5 min, 35 cycles of 30 s at 95 °C, 45 s at 63 °C, 45 s at 72 °C, and a final elongation step for 5 min at 72 °C. Amplified PCR product was purified and thereafter directly sequenced (Integrated DNA Technologies/1st BASE, Singapore).

### Statistical analysis

Statistical analysis and graphical presentation were carried out using GraphPad Prism® 7.02.

## Results

### Adult *Ae. aegypti* resistance development to tested insecticides

**Fig. 2:**
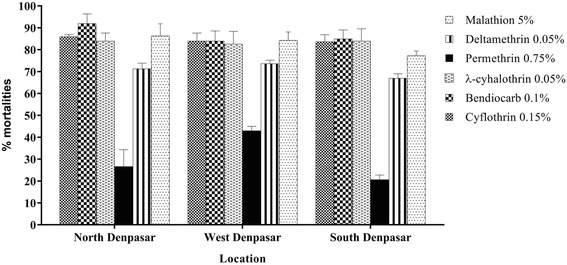
Resistance profiles to different insecticides tested in *Ae. aegypti* from Denpasar, Bali, Indonesia. The bars are percent mortalities after exposure to insecticides with error bars representing standard deviations

A total of 1,890 specimens were tested against various insecticide-impregnated papers according to WHO protocols [26]. All tested *Ae. aegypti* mosquitoes from urban locations presented resistance levels to insecticides as shown in Fig. 2. The mortality percentages obtained for these tested mosquitoes showed that all mosquito populations collected in Denpasar were resistant to tested insecticides according to WHO criteria [26], i. e. with mortalities < 90%. Mosquitoes from southern Denpasar showed the highest resistance development to malathion 5% which was significantly different from the resistance observed in northern Denpasar (*t* = 2.81, *P* = 0.048) but no significant differences were observed when compared to the western parts of Denpasar (*t* = 2.65, *P* = 0.057). Resistance development to deltamethrin 0.05% was not significantly different between these urban areas. Moreover, permethrin resistance was quite high with less than 50% mosquito mortalities in all examined areas. Mosquitoes from southern and northern Denpasar showed the highest levels of resistance which differed significantly from that in mosquitoes from western Denpasar (*t* = 13.4, *P* ≤ 0.001). Furthermore, mild *Ae. aegypti* resistance development, with approximately 80–90% mortalities, were also observed against λ-cyhalothrin 0.05%, bendiocarb 0.1% and cyflothrin 0.15%, respectively.

### Sequencing and mosquito genotyping the voltage-gated sodium channel gene (*Vgsc*)

Sequencing of domain II *Vgsc* gene was analyzed to identify possible S989P and V1016G allele variations within collected mosquito population. Mutations of S989P were found in mosquitoes originating from Denpasar. S989P allele variations were found almost equally in wild type-, heterozygote- and homozygote-specimens. Frequency of P allele was half of resistant phenotypes to permethrin (Table 2). The electropherogram pattern of sequenced domain did not show any separate C encoding prolin pattern in all examined samples. Nonetheless, the presence of T encoding for serin in wild type or heterozygote forms was quite clear. G allele was a dominant allele found in the population with 0.7 frequency of resistance and 0.5 in succeptible groups. Clear electropherograms were always found in the homozygote samples. The homozygote G1016G was the highest allele to be found in the resistance group in comparison to S989P and F1534C (Table 2). Exemplary amplicons representing V1016G and S989P polymorphisms, respectively, were deposited in GenBank (accession nos. KY057038 and KY057037).

**Table 2:** Mutation of S989P, V1016G and F1534C among *Ae. aegypti* mosquitoes in Denpasar, Bali

Phenotypes	No. of samples	Alleles						Allele frequency (%)
		*n*	F (%)	*n*	F (%)	*n*	F (%)	
		SS		SP		PP		P allele
Alive	65	22	0.34	20	0.31	23	0.35	0.51
Dead	45	27	0.60	15	0.33	3	0.07	0.23
		VV		VG		GG		G allele
Alive	70	10	0.14	22	0.31	38	0.54	0.70
Dead	62	20	0.32	22	0.35	20	0.32	0.50
		FF		FC		CC		C allele
Alive	52	28	0.54	11	0.21	13	0.25	0.36
Dead	45	34	0.76	5	0.11	6	0.13	0.19

Allele-specific PCR of F1534C showed distributions F to C point mutations in the domain III of *Vgsc* gene. In the resistance (alive) phenotypes, homozygote frequencies of CC were 0.21 and heterozygote frequencies of FC were 0.25. In contrast, succeptible (dead) mosquito samples showed CC 0.13 and FC 0.11 frequencies. Allele containing C was found in heterozygotes and CC allele was relatively rarely found in the population of susceptible samples. Total C frequency was lower when compared to F frequency in Denpasar mosquitoes (Table 2). Sequences of domain III showed F1534C mutations and wild-type F1534F mutations, which were also deposited in GenBank (accession nos. KY078304 and KY078303).

When calculated separately, odd ratio (OR) S989P of samples (*n* = 110) showed association with resistance to permethrin (Fisher’s exact test, *P* = 0.0005, OR = 7.67, 95% CI = 2.14–27.49). Further, V1016G (*n* = 132) also showed significant correlation with resistance to permethrin (Fisher’s exact test, *P* = 0.014, OR = 2.49, 95% CI = 1.23–5.07). Conversely, F1534C mutation (*n* = 97) showed no significant association with phenotypes tested for permethrin (*P* ≥ 0.05). However, when C was present with P and G heterozygotes (Table 3, no. 2) and homozygotes (Table 3, no. 14), all mosquitoes (*n* = 9) were identified as totally resistant (alive) to permethrin 0.75%. Due to our limited data (results included only 97 clearly identified homozygote and heterozygote patterns with three point mutations), alleles presented in Table 3 only give a tendency in synergistic pattern of resistance. Strong association of resistance showed by PVF/PGF, OR of 15, CI: 1.21–185.21 (Table 3, no. 8), may indicate synergistic resistance by simultaneous presence of P and G.

**Table 3:** Alleles and their association with resistance to permethrin of mosquitoes from Denpasar, Bali

Number	Allele variation	Phenotypes		Total	OR		
		Dead (susceptible)	Alive (resistant)		OR	Lower limit	Upper limit
1	SVF	9	3	12			
2	SVF/PGC	0	4	4	inf	–	–
3	SVF/PGF	3	3	6	3	0.3801	23.6801
4	SGF/PGF	4	2	6	1.5	0.1761	12.7759
5	SVC	2	1	3	1.5	0.0975	23.0705
6	SGF	6	5	11	2.5	0.4279	14.607
7	PGF/PGC	0	3	3	inf	–	–
8	PVF/PGF	1	5	6	15	1.2149	185.2069
9	PVF/PVC	0	1	1	inf	–	–
10	SGC	0	4	4	inf	–	–
11	SVF/SGF	10	5	15	1.5	0.2765	8.1383
12	SGF/PGC	0	1	1	inf	–	–
13	SGF/SGC	2	1	3	1.5	0.0975	23.0705
14	PGC	0	5	5	inf	–	–
15	PGF	1	1	2	3	0.14	64.2651
16	PVF	0	1	1	inf	–	–
17	PVF/PGC	0	1	1	inf	–	–
18	SGC/PGC	1	2	3	6	0.3901	92.282
19	SGF/PGF	0	2	2	inf	–	–
20	SGP/PGC	0	2	2	inf	–	–
21	SVF/SVC	1	0	1	0	–	–
22	SVF/PVC	2	0	2	0	–	–
23	SVF/PVF	2	0	2	0	–	–
24	PVC/PGC	1	0	1	0	–	–
Total		45	52	97			

*Abbreviations*: *inf* infinity number (OR for these points are difficult due to the presence of 0 numbers for statistical analysis)

## Discussion

*Aedes* mosquito control still challenges public health authorities of subtropical and tropical countries worldwide due to mosquito ability to adapt fast to adverse breeding conditions and also by the development of resistance against routinely used insecticides [7, 10, 12, 14, 27, 28]. Moreover, the capability of *Aedes* spp. to transmit a wide spectrum of relevant arthropod-borne diseases in the subtropics and tropics makes this genus a serious threat to human health. *Aedes* control through the usage of insecticides is the preferred choice by the Indonesian government to reduce the number of DF. Insecticide fogging is a direct action program performed by Indonesian public health agencies when human DF cases are reported to occur within national territories.

Global movement of humans, international transportations, poor housing conditions, tourism and breeding opportunities of mosquitoes are well-known risk factors to be involved in the epidemiology and transmission of vector-borne diseases, particularly in underdeveloped geographic regions [29]. The above mentioned risk factors increase widespread use of insecticides and consequent resistance development within exposed mosquito populations. Besides, several complex mechanisms also contribute to serious limitations to the long-term usage of insecticides due to efficacy reduction [30]. Resistance indicators of mosquitoes can be analyzed in various ways; however, metabolic resistance-based methodologies are more costly in comparison with gene-targeted resistance-associated assays.

Human DF cases in the city of Denpasar, Bali, reached 6,898 with 38 mortalities recorded from 2014 until mid 2016 according to data records [Bali Health Agency (BHA), Indonesia]. Insecticide fogging with pyrethroid- and organophosphate-based compounds have been performed 818 times covering all regions of Denpasar city in those 3 years, i. e. 255 times in 2014; 310 in 2015; and 253 until mid 2016 (BHA, Indonesia). These data also showed trends of fogging intensities annually in the city of Denpasar. Fogging activities mainly targeted high burden areas based on patients reported from local hospitals. Moreover, urban areas with touristic attractions used mosquitoes repellents at higher frequencies in order to avoid mosquito bites. The commercial brand of repellents based on pyrethroid compounds were used by almost all citizens of Denpasar (BHA, Indonesia).

To our knowledge, we here present for the first time indications of resistence development in *Ae. aegypti* populations against commonly used insecticides regularly applied in control programs in the city of Denpasar, Bali. The detailed molecular *Ae. aegypti* phenotype analyses showed high resistance patterns to all insecticides tested. No significant differences from North, West and South Denpasar were detected pointing that insecticide compounds may have been used continuously in almost all areas with the same frequency and/or due to the fact that investigated areas were all from one city district. Noticeably, mosquito repellents such as skin lotions, in-house sprays and coils, are still broadly used by local citizens as well as by international travellers. Thus, mosquito exposure to such various repellent ingredients is continuous and even higher in this touristic tropical island when compared to less tourist-visited Indonesian islands. Our *Ae. aegypti* resistance results corroborate previous mosquito resistance findings in Java [17, 18], an adjacent island to Bali, which showed similar resistance patterns in *Ae. aegypti* populations. Although the analyzed area in these studies was much larger than that in our study [17], the resistance patterns were almost the same, thereby suggesting similar usage of insecticide active ingredients in Java. However, multiple insecticide resistance can also be transmitted from dry eggs stuck on containers which are transported from one area to another by humans. Although male or female mating competitiveness of resistant insect species are under investigation [31, 32], it is of concern that with or without insecticide exposure resistance can be accelerated through mating processes when insecticide resistance is initially detected as reported previously elsewhere [29, 30].

Based on analysis pointing on *kdr* mutations, V1016G haplotypes were most frequently found in comparison with the two other analyzed targets, i. e. F1534C and S989P. This haplotype is commonly found in *Ae. aegypti* [6, 13, 16, 17, 33] but not in South America where the haplotype V1016I is mainly dominating [34]. Conversely, the haplotype F1534C is much less frequently found in *Aedes* populations when compared to V1016G and S989P genes. This is in line with previous reports of Java [17, 18], although it is known that F1534C also significantly contributes to type I phyrethroid resistance as published elsewhere [35] and was also found as major allele in *Aedes* mosquitoes of South America [10].

Sequencing results of domain II revealed that allele carying 2 mutants on S989P and V1016G, respectively, appeared in relatively high numbers of resistant *Ae. aegypti* mosquitoes. Although the significant contribution on resistance development towards pyrethroids of S989P allele is still under debate, it may play an additive role in *Ae. aegypti* insecticide resistance development [16, 24, 28], in which the V1016G allele seems to play a pivotal role. Phenotypes in Table 3 may not only be explained by *kdr* mutations representing insensitivity of target sites towards tested insecticide as metabolic capacities within the genus *Aedes* might also explain phenotype variations. Consistently, wild-type genotypes with resistant phenotypes have been reported to occur suggesting enhanced metabolic detoxification capacities by catalytic sites of certain mosquito enzymes [36].

Periodical surveillance of insecticide resistance development in mosquitoes will clearly contribute in setting appropriate goals and evaluation process of ongoing control programs by Indonesian governmental authorities. Initial detection of resistance development may contribute to generate proper management/control strategies such as insecticide rotation programs or the combination of different ingredient types of insecticides in order to prolong chemical efficacy in combating these vectors.

## Conclusions

Regular surveillance surveys on insecticide resistance development in *Ae. aegypti* mosquitoes will help local public health authorities to set better goals and allow proper evaluation of ongoing mosquito control strategies. Initial detection of insecticide resistance will also contribute to conduct proper actions in delaying mosquito resistance development such as insecticide rotation or combination of compounds in order to prolong chemical efficacy in combating *Ae. aegypti* vectors in Indonesia.

## Abbrevations

DF: Dengue fever
IST: Insecticide succeptibility test
*Kdr*: Knockdown resistance
OR: Odds ratio
RT: Room temperature
Vgsc: Voltage-gated sodium channel gene
WN: West Nile
